# A Study on the Application of Recombinant Factor C (rFC) Assay Using Biopharmaceuticals

**DOI:** 10.3390/microorganisms12030516

**Published:** 2024-03-04

**Authors:** Da Hee Kang, Song Yeol Yun, SoYoung Eum, Kyung Eun Yoon, Seung-Rel Ryu, Chulhyun Lee, Hye-Ryeon Heo, Kwang Moon Lee

**Affiliations:** Biologics Research Division, Pharmaceutical and Medical Device Research Department, National Institute of Food and Drug Safety Evaluation, Cheongju 28159, Republic of Korea; rkdekgml12@korea.kr (D.H.K.); songyoul90@korea.kr (S.Y.Y.); syeum10@korea.kr (S.E.); ivysponge@korea.kr (K.E.Y.); srryu@korea.kr (S.-R.R.); ph1025@korea.kr (C.L.); heohr@korea.kr (H.-R.H.)

**Keywords:** bacterial endotoxin testing, endotoxin, *Limulus* amebocyte lysate, recombinant factor C

## Abstract

Gram-negative bacterial endotoxins can cause pathophysiological effects such as high fever when introduced into the bloodstream. Therefore, endotoxin testing is necessary when producing injectable pharmaceuticals. The pharmaceutical industry has widely used *Limulus* amebocyte lysate (LAL) to certify product quality. However, ethical concerns have been raised and the increasing scarcity of *Limulus polyphemus* necessitates the development of novel testing techniques. Recombinant factor C (rFC) was developed using genetic engineering techniques. The aim of this study was to investigate the validity of rFC testing and compare it with the LAL method. The specificity, linearity, accuracy, precision, and robustness of the rFC assay were evaluated. After validation, the rFC assay was found to be suitable for endotoxin detection. We compared the accuracy of the rFC and LAL assays using reference standard endotoxin. The rFC assay was as accurate as the LAL assay. We also compared the two assays using biopharmaceuticals. Greater interference occurred in some samples when the rFC assay was used than when the LAL assay was used. However, the rFC assay overcame the interference when the samples were diluted. Overall, we suggest that rFC can be applied to test biopharmaceuticals.

## 1. Introduction

Endotoxins (e.g., lipopolysaccharide, LPS) are present in the outer membrane of all Gram-negative bacteria and can cause toxic effects such as fever, septic shock, and multiorgan failure by producing proinflammatory cytokines and activating the coagulation cascade [1]. Therefore, elimination of endotoxins in final pharmaceutical products is an important challenge for manufacturers [2]. The *Limulus* amebocyte lysate (LAL) test is commonly used to detect endotoxins. The underlying mechanism of this method is based on the defense system of horseshoe crab hemocytes. When exposed to endotoxins, factor C is activated, and the coagulation cascade is initiated. Eventually, a coagulin gel is formed that traps the invading bacteria [3,4]. However, LAL contains many other proteins; thus, the coagulation cascade can be triggered by glucan, which is not pyrogenic, thereby producing false-positive endotoxin results [5,6].

Major issues with the LAL method related to ethical concerns and limited supplies have been raised. For example, the process of bleeding horseshoe crabs is associated with crab mortality rates of up to 30% [7]. To address these challenges, a recombinant factor C (rFC) assay was developed as an alternative to LAL. The cDNA sequence of factor C was cloned from the mangrove horseshoe crab (*Carcinoscorpius rotundicauda*) and expressed in several hosts by Ding et al. in 1997 [8,9,10]. In the rFC assay, the synthetic rFC molecule is activated by an endotoxin, and amino-methylcoumarin, a fluorogenic substrate, is then cleaved to yield a fluorogenic compound [11,12,13]. Because rFC is biotechnologically engineered, the rFC-based assay has high purity, endotoxin specificity, and low lot-to-lot variation [14,15]. In addition, the rFC assay does not contain the factor G protein; thus, it cannot lead to false positives or result in enhancement of β-glucan [16]. Recombinant proteins can be easily and sustainably produced in unlimited amounts without using animals [17]. In recent years, many studies on validation of the rFC assay and comparability of the LAL and rFC assays have been conducted [14,15,16,17].

rFC assays are now available from many manufacturers and suppliers. rFC assay was listed as a compendial method in European Pharmacopoeias [18]. Considering the need to examine whether rFC assay could be used as an alternative, in this study, we validated the rFC assay in compliance with the International Council on Harmonization of Technical Requirements for Registration of Pharmaceuticals for Human Use guidelines and Bioanalytical Method Validation Guidance or Industry issued by the Food and Drug Administration. We also compared the LAL and rFC assays using reference standard endotoxin (RSE) and biopharmaceuticals to determine the applicability of the rFC assay.

## 2. Materials and Methods

### 2.1. Reagents

LAL reagent water (W110), Control Standard Endotoxin (CSE) 10 ng/vial (E120), Kinetic Turbidimetric LAL 5.2 mL (R19000), Kinetic Chromogenic LAL 3.2 mL (R17100), and 96-well microplates (Falcon 353072, Becton Dickinson, Franklin Lakes, NJ, USA) were all purchased from Charles River Laboratories (Wilmington, MA, USA) and used for the LAL test. Endozyme II (890030), Endozyme II GO (890031), and Endolisa (609033), all purchased from bioMérieux (Marcy-l’Étoile, France), and PyroGene (50-658U) purchased from Lonza (Basel, Switzerland), were used for the rFC test. We used 96-well microplates provided with each kit from bioMérieux and a 96-well flat clear-bottomed black polystyrene microplate (3603, Corning, New York, NY, USA). All assays were performed using pyrogen-free tips and glass tubes.

### 2.2. Bacterial Endotoxin Test (BET)

#### 2.2.1. LAL Assays

The kinetic chromogenic assay (KCA) and kinetic turbidimetric assay (KTA) reagents were used for the LAL assay. The LAL assays were performed according to the manufacturer’s instructions and analyzed using an ELx808IU absorbance microplate reader (BioTek Instruments GmbH, Bad Friedrichshall, Germany) with Endoscan V software version 6.0.2 (Charles River Laboratories). The temperature of the instrument incubator was maintained at 37 °C during the measurement. Absorption was observed at 405 nm with an onset optical density of 0.1 for KCA and at 340 nm with an onset optical density of 0.05 for KTA.

#### 2.2.2. rFC Assays

The rFC assays were performed according to the manufacturer’s instructions. Assays with rFC reagent from Biomerieux were analyzed using a BioTek Synergy HTX fluorescence reader (Biotek, Winooski, Vermont, USA) with ENDONEXT software version 1.1 (bioMérieux, Marcy-l’Étoile, France). Samples were preincubated in the plate reader at 37 °C for 5 min before adding the working reagent (a mixture of fluorogenic substrate, assay buffer, and the enzyme solution). Thereafter, the plates were measured with excitation/emission wavelengths at 380/445 nm and incubated at 37 °C during the measurement. Assays with rFC reagent from Lonza were analyzed using a BioTek FLx800 Fluorescence reader (Biotek, Winooski, Vermont, USA) with WinKQCL software version 6.0.1 (Lonza, Basel, Switzerland). The plate was preincubated in the plate reader at 37 °C for 10 min, and then the working reagent was dispensed to each well. The plate was incubated at 37 °C during the measurement, and the fluorescence was read with excitation/emission wavelengths at 380/440 nm.

### 2.3. Validation of rFC Assay

To detect endotoxins, an rFC-based assay was conducted using Endozyme II. Standard curves were generated by using diluted solutions prepared through repeated 1:10 dilutions of CSE stock solution (50 EU/mL) and via linear regression analysis. BET was carried out using four replicates of sample, half of which were spiked samples. CSE was added to the sample at a final concentration of 0.5 EU/mL The spiked samples were in a positive product control (PPC) group and prepared to ensure the presence of interference.

#### 2.3.1. Linearity

Four concentrations of CSE (0.005, 0.05, 0.5, and 5 EU/mL) were prepared in duplicate for the standard curve. The acceptance criterion for linearity was a correlation coefficient (|R|) greater than 0.9800 for the standard curve.

#### 2.3.2. Accuracy

Four endotoxin concentrations (0.05, 0.1, 0.5, and 1.0 EU/mL) were tested within the standard curve range. Accuracy was evaluated based on sample recovery (%), which should be between 50% and 200%.

#### 2.3.3. Precision

Four endotoxin concentrations (0.05, 0.1, 0.5, and 1.0 EU/mL) were tested. Precision was assessed using the coefficient of variation (CV) obtained from repeated measurements of an assay (repeatability), analysts (intermediate precision), and laboratories (reproducibility). We assessed the CV (%) of both sample recovery and PPC recovery for precision. The acceptance criterion for precision and robustness was CV < 25%.

#### 2.3.4. Robustness

Four endotoxin concentrations (0.05, 0.1, 0.5, and 1.0 EU/mL) were tested. Robustness was confirmed by the CV (%) obtained from the results of different reagent lots. We assessed the CV (%) of both sample recovery and PPC recovery. The acceptance criterion for precision and robustness was CV < 25%.

### 2.4. Comparison of LAL and rFC Test Methods

To compare the LAL and rFC assays, we used KCA and KTA reagents and Endozyme II, Endozyme II GO, Endolisa, and PyroGene kits. For each assay, a standard curve was prepared from 0.005–5 EU/mL CSE (fresh CSE for each assay) and generated using a linear regression model. RSE (10/178, NIBSC) was used as the test sample. Five RSE concentrations (1, 0.5, 0.1, 0.05, and 0.01 EU/mL) were diluted with endotoxin-free water. For each of the six types of assay, CSE was added to the PPC group so that the final endotoxin concentration of CSE was 0.5 EU/mL. Unlike the other reagents, the standard curve of Endolisa was 0.05–50 EU/mL and fitted using a 4-parameter logistic regression model. Therefore, four different RSE concentrations (5, 1, 0.5, and 0.1 EU/mL) were tested. All samples were measured in duplicate, and the means of sample recovery (%) and percentage difference were calculated for each reagent. The values for all reagents were compared using the Kruskal–Wallis H test, and then Dunn’s test was performed for the post hoc analysis. The values of the LAL and rFC assays were compared using the Wilcoxon rank-sum test.

### 2.5. Application of rFC Assay to Biopharmaceuticals

BET was conducted using Endozyme II, PyroGene, KTA, and KCA kits. Fourteen recombinant products, including antibody therapeutics (Ab) and recombinant protein products (P), two botulinum toxin products, and 14 vaccines, including a bacterial (BV) and viral (VV) vaccine, were used as test samples. The samples were diluted with endotoxin-free water, and the dilution factors were determined using an inhibition/enhancement test. CSE was added to the PPC group so that the final endotoxin concentration was 0.5 EU/mL. As the PPC recovery must be within 50–200%, serial dilution was performed to not exceed the maximum valid dilution (MVD). Four different testing methods were used to evaluate each biopharmaceutical type. Each test was performed once, and three sets of duplicate samples were prepared for each assay.

## 3. Results

### 3.1. Validation of rFC Assay

#### 3.1.1. Linearity

Linearity was analyzed based on the results obtained using the four CSE concentrations. A standard curve was calculated using a regression model by fitting a linear model log(Y) = Alog(X) + B. The |R| values of assays 1, 2, and 3 were 1.000, 0.9991, and 0.9999, respectively, which met the target criterion (|R| ≥ 0.980).

#### 3.1.2. Accuracy

Four different concentrations were prepared with three sets of duplicate samples, and three assays were conducted by a single analyst. The actual endotoxin levels in the samples were compared with the expected CSE concentrations. The sample recoveries (%) were 97.4–121.0%, which was within the acceptable range of 50–200% (Table 1).

#### 3.1.3. Precision (Repeatability)

One analyst performed three assays, and three sets of duplicate samples were measured in each assay. The CV (%) of the actual endotoxin concentration and PPC recovery (%) among the three sets was calculated. All CV results (%) were within 0.6–11.2%, and within the acceptance criterion of <25% (Table 2).

#### 3.1.4. Precision (Intermediate)

Two analysts each performed all three assays. Three duplicate samples were prepared for each assay. The average endotoxin concentration and PPC recovery (%) were calculated for each assay. The CV (%) values obtained by the two different analysts were within 3.6–6.1% and 2.2–14.8% for the actual endotoxin concentration and PPC recovery (%), respectively. All CV results (%) met the acceptance criterion of <25% (Table 3).

#### 3.1.5. Precision (Reproducibility)

Three different laboratories performed all three assays. Four different concentrations of CSE were prepared in duplicate. The CV (%) of the actual concentration and PPC recovery (%) between laboratories was within 4.7–12.2% and 5.5–14.3%, respectively. All CV results (%) met the acceptance criterion of <25% (Table 4).

#### 3.1.6. Robustness

Robustness was assessed using the CV (%) obtained from three different reagent lots. The CV (%) of the actual endotoxin concentration and PPC recovery (%) were 5.6–10.7% and 4.7–7.9%, respectively. All CV (%) results met the acceptance criterion of <25% (Table 5).

### 3.2. Comparison of LAL and rFC Test Methods

The rFC (Endozyme II, Endozyme II GO, Endolisa, and PyroGene) and LAL (KCA and KTA) reagents were used for equivalence testing. The rFC reagents were labeled A–D. Five different concentrations (1, 0.5, 0.1, 0.05, and 0.01 EU/mL) of RSE were used. Each sample was prepared in duplicate, and the average sample recovery (%) and percentage difference for each reagent were calculated (Table 6).

**Figure 1:**
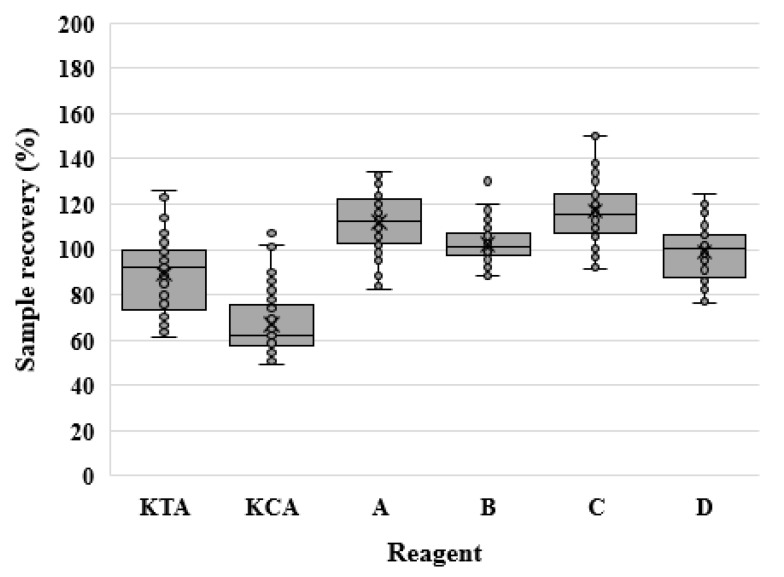
Sample recovery (%) for LAL and rFC assays. The sample recovery (%) for each reagent is graphically represented using a box plot. LAL reagents contain KTA and KCA, and rFC reagents are labeled A–D.

Only data that met the inclusion criteria were used in the statistical analysis. Figure 1 shows the data distribution and outliers. The sample recovery (%) increased in the order KCA < KTA < D < B < A < C. However, there was no significant difference between reagents B and D or A and C.

We also analyzed the absolute value of the difference between sample recovery (%) and 100%. The percentage difference was higher in the order of B < D < A < KTA < C < KCA. When the results of the percentage difference were statistically analyzed using the Kruskal–Wallis H test, the reagents showed a significant difference (*p* < 0.001). Then, Dunn’s tests were performed for the post hoc analysis. No significant difference was found among KTA, A, and C; however, KCA showed significant differences compared with all rFC reagents. The mean percentage difference of the rFC assay was significantly lower (*p* < 0.001) than that of the LAL assay in Wilcoxon rank-sum test analysis when the reagents were grouped according to the test method (LAL and rFC) (Table 7).

**Figure 2:**
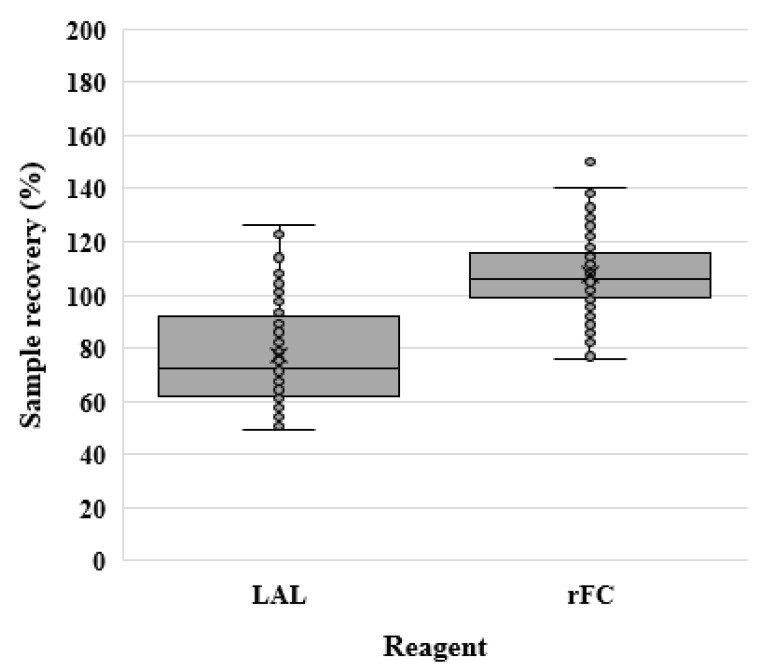
Sample recovery (%) for the LAL and rFC assays. The sample recovery (%) for each reagent is graphically represented using a box plot.

The data distribution and outliers are shown in Figure 2.

### 3.3. Application of rFC Assay to Biopharmaceuticals

We compared the LAL and rFC test methods using biopharmaceuticals including vaccines, recombinant products, and toxin products. An inhibition/enhancement test was performed on each product to determine the appropriate dilution factor. Each diluted product was analyzed in three sets of duplicate samples. All recombinant products and toxin products had the same dilution factors regardless of the assay type, whereas some bacterial (BV4 and BV6) and viral (VV2, VV3, VV4, VV5, and VV6) vaccines needed additional dilution when using the rFC assay compared to when using the LAL assay to overcome interference. Furthermore, rFC reagents from two different manufacturers had different dilution factors for BV4, VV4, and VV5, although they were used in the same rFC-based assay. None of the biopharmaceuticals exceeded the endotoxin limits, and the average PPC recovery (%) values of the samples were within 50–200% (Table 8, Table 9 and Table 10).

## 4. Discussion

Over the past five decades, horseshoe crab hemolymph has been used as a source of lysate reagents for endotoxin detection and quantification. However, this dependence on horseshoe crabs has led to limited reagent supplies owing to decreases in the horseshoe crab population [4]. Ethical issues surrounding the continued use of animals have also brought global attention to the need for alternatives to animal sources. Therefore, the rFC assay has been proposed as an alternative to LAL.

We determined the linearity, accuracy, precision, and robustness of the rFC assay through method validation. The rFC assay satisfied the acceptance criteria for all parameters. The results indicated that the rFC assay is reliable for endotoxin detection. We then compared the LAL and rFC assays using RSE and biologics. We tested five concentrations (1, 0.5, 0.1, 0.05, and 0.01 EU/mL) of RSE to investigate the accuracy of the assays. All rFC reagents were substantially different from the LAL reagents when sample recovery (%) was statistically analyzed. The sample recovery of the rFC reagents was 98.9–116.9%. The sample recoveries of all rFC reagents were closer to 100% than the sample recovery (%) of KCA. Moreover, Piehler et al. demonstrated that rFC assays are accurate, with sample recoveries of nearly 100% [17]. The percentage difference of all rFC reagents, except for reagent C, was lower than that of KTA. In addition, no significant difference was found in percentage difference between KTA and rFC reagents A and C, although the average sample recovery (%) using KTA was less than 100%, whereas those using A and C were more than 100%. Among all reagents, rFC reagent B showed the lowest percentage difference, whereas the percentage difference of KCA was the highest. Overall, our results demonstrated that the rFC test method is as reliable as the LAL method.

We also investigated the performance of the rFC assay for the endotoxin test of biopharmaceuticals and compared it with that of the LAL assay. Generally, in bacterial endotoxin test, pH, salt, detergents, chelating agents, and proteins are potential influencing agents [19]. Therefore, adjusting pH, diluting samples, or neutralizing the influencing agent is required to overcome product interference. In this study, interference was overcome by diluting the samples with endotoxin-free water within the MVD. Then, we compared the sample dilution factors and PPC recovery (%) results obtained from each reagent. Many recombinant products contain polysorbates, which may cause false-negative results [20,21]. However, our results showed that there was no severe interference with the PPC recovery (%), because all the samples, except for P2 and P4, were diluted less than 50-fold. This is probably owing to the small amount of polysorbate in the products. Moreover, the concurrent existence of a chelator and nonionic surfactant is enough to reduce the ability of endotoxin detection. However, the existence of only one of the formulation constituents does not cause a remarkable disruption in endotoxin recovery [22]. Any interferences in P2 and P4 were probably caused by components other than polysorbate, which were also overcome through further dilution. Toxin products also showed no severe interference because the samples were only diluted 10-fold. However, some vaccines contained different dilution factors for each reagent. When using rFC rather than LAL, more interference occurred in some of the samples containing aluminum hydroxide or amorphous aluminum hydroxyphosphate sulfate. Aluminum hydroxide interfered greatly with both KCA and KTA assays [23]. Polymers in metal are used as carriers in complicated pharmaceutical formulations; however, the structure and size of endotoxin can also be interfered with by aluminum salt [24]. We identified that the rFC assay is also susceptible to interference by aluminum, as the LAL assay is; however, the problem of interference may be reduced as the rFC assay can withstand more dilution than the LAL assay.

All biopharmaceuticals, including recombinant products, toxin products, and vaccines, satisfied the criteria. However, we found some samples with different dilution factors between reagents or differences in PPC recovery (%) between reagents, although an identical dilution factor was used for the samples. In the case of LAL assays, KTA is linked to the rate of turbidity, and KCA uses a synthetic chromogenic substrate. The rFC assay detects endotoxins using fluorescence. Thus, differences may occur between assays. However, there were also considerable differences in the PPC recovery (%) of some products between rFC reagents A and B, even though they used the same rFC-based assay. Specifically, P4 showed the largest difference in PPC recovery (57%) between reagents A and B. Furthermore, BV4, VV4, and VV5 showed a 100-fold difference in dilution factor when using the rFC reagents A and B. Minimum dilution factors of injectable drugs differ by at least two- to fourfold between PyroGene and EndoZyme II [16]. We assumed that the difference in the manufacturing process or combination ratio of working reagents of rFC reagents A and B induced the disparity in the results of PPC recovery (%).

In summary, we identified that the rFC is reliable for detecting endotoxins through the validation of the rFC assay and comparison with the LAL assay using RSE. Our results provide new evidence for using rFC assay in replacement of laboratory animals. Moreover, our data demonstrated that the rFC assay would be applicable to biopharmaceuticals because it met the acceptance criteria for PPC recovery (%) by diluting samples within the MVD. However, we also encountered some interference. In this study, we overcame the interference by diluting samples with water alone. However, buffers such as saline can also be used to prevent interference with the LAL assay [25]. Further studies are required to investigate other methods for reducing the dilution factor as an alternative to diluting samples with water in cases in which severe interference with the rFC assay is encountered. In conclusion, our data suggest that the rFC assay is accurate and reliable for detection of endotoxins and can be used in testing biopharmaceuticals. Also, when the rFC assay is applied to products in the biopharmaceutical industry, the validation should be adequately performed, taking the components of the product into consideration.

## Figures and Tables

**Table 1:** Sample recovery (%) of three assays.

Endotoxin Concentration (EU/mL)	Accuracy (%)										
	Assay 1			Assay 2			Assay 3			Mean	CV (%)
1.0	109.7	108.2	107.7	109.8	110.1	111.6	101.5	101.8	103.3	107.1	3.6
0.5	97.4	98.6	97.4	100.6	99.6	102.8	102.2	101.6	102.8	100.3	2.2
0.1	105.0	105.0	104.0	118.0	121.0	121.0	99.0	101.0	101.0	108.3	8.3
0.05	108.0	110.0	108.0	116.0	118.0	118.0	100.0	100.0	102.0	108.9	6.7

**Table 2:** CV (%) of the actual concentration and PPC recovery from different assays.

Endotoxin Concentration (EU/mL)	CV (%) of Actual Concentration			
	Assay 1	Assay 2	Assay 3	Mean
1.0	1.0	0.9	0.9	0.9
0.5	0.7	1.6	0.6	1.0
0.1	0.6	1.4	1.2	1.0
0.05	1.1	1.0	1.1	1.1
Endotoxin Concentration (EU/mL)	CV (%) of PPC Recovery			
	Assay 1	Assay 2	Assay 3	Mean
1.0	11.2	10.0	8.8	10.0
0.5	2.0	6.3	4.2	4.2
0.1	0.9	3.4	2.7	2.3
0.05	1.8	3.7	0.9	2.1

**Table 3:** Results of actual concentration and PPC recovery (%) from two different analysts.

Endotoxin Concentration (EU/mL)	Actual Concentration (EU/mL)						
	Analyst 1			Analyst 2			CV (%)
	Assay 1	Assay 2	Assay 3	Assay 1	Assay 2	Assay 3	
1.0	1.085	1.105	1.020	0.961	1.072	1.051	5.0
0.5	0.489	0.505	0.510	0.517	0.534	0.539	3.6
0.1	0.105	0.120	0.100	0.111	0.110	0.108	6.1
0.05	0.054	0.059	0.050	0.055	0.055	0.056	5.3
Endotoxin Concentration (EU/mL)	PPC Recovery (%)						
	Analyst 1			Analyst 2			CV (%)
	Assay 1	Assay 2	Assay 3	Assay 1	Assay 2	Assay 3	
1.0	111.4	109.9	101.1	75.3	91.9	85.9	14.8
0.5	91.2	93.3	99.8	93.6	85.0	89.6	5.3
0.1	98.4	112.6	103.7	98.8	100.1	93.5	6.4
0.05	99.1	101.7	102.9	98.1	98.8	97.1	2.2

**Table 4:** Results of actual concentration and PPC recovery (%) from three different laboratories.

Endotoxin Concentration (EU/mL)	Actual Concentration (EU/mL)									
	Laboratory 1			Laboratory 2			Laboratory 3			CV (%)
1.0	1.097	1.098	1.015	1.087	1.014	1.084	0.962	1.016	1.020	4.7
0.5	0.487	0.503	0.511	0.560	0.511	0.593	0.477	0.507	0.503	7.1
0.1	0.105	0.118	0.099	0.119	0.110	0.119	0.114	0.103	0.096	8.1
0.05	0.054	0.058	0.050	0.060	0.050	0.064	0.069	0.054	0.049	12.2
Endotoxin Concentration (EU/mL)	PPC Recovery (%)									
	Laboratory 1			Laboratory 2			Laboratory 3			CV (%)
1.0	97.2	97.7	90.9	104.2	85.3	81.0	61.3	81.8	88.2	14.3
0.5	90.7	90.0	98.6	107.3	89.4	93.4	67.5	84.6	91.0	11.9
0.1	98.8	109.2	100.7	111.4	102.6	102.0	90.0	95.7	100.1	6.4
0.05	99.5	100.0	102.9	104.5	106.2	102.5	88.4	94.1	100.3	5.5

**Table 5:** Actual concentration and PPC recovery (%) from different lot numbers.

Endotoxin Concentration (EU/mL)	Actual Concentration (EU/mL)									
	Lot 1			Lot 2			Lot 3			CV (%)
1.0	1.085	1.105	1.020	1.039	1.078	1.108	0.981	1.195	1.073	5.7
0.5	0.489	0.505	0.510	0.503	0.555	0.515	0.501	0.574	0.549	5.6
0.1	0.105	0.120	0.100	0.093	0.122	0.098	0.105	0.117	0.107	9.5
0.05	0.054	0.059	0.050	0.045	0.063	0.049	0.053	0.060	0.053	10.7
Endotoxin Concentration (EU/mL)	PPC Recovery (%)									
	Lot 1			Lot 2			Lot 3			CV (%)
1.0	111.4	109.9	101.1	118.1	96.5	110.8	91.7	109.9	102.2	7.9
0.5	91.2	93.3	99.8	107.0	97.5	105.4	95.4	104.1	99.2	5.5
0.1	98.4	112.6	109.7	106.5	104.2	106.6	104.7	108.0	97.4	4.7
0.05	99.1	101.7	102.9	103.3	105.1	106.2	102.5	107.5	89.6	5.2

**Table 6:** Sample recovery (%) and percentage difference of LAL and rFC reagents.

Parameter	Reagent	n	Mean
Sample recovery (%) (a < b < c < d)	LAL (KTA)	68	89.07 ^b^
	LAL (KCA)	78	67.03 ^a^
	rFC (A)	81	111.84 ^d^
	rFC (B)	75	102.25 ^c^
	rFC (C)	60	116.89 ^d^
	rFC (D)	48	98.93 ^c^
Percentage difference (a < b < c < d)	LAL (KTA)	68	15.36 ^c^
	LAL (KCA)	78	33.31 ^d^
	rFC (A)	81	14.57 ^c^
	rFC (B)	75	5.87 ^a^
	rFC (C)	60	17.78 ^c^
	rFC (D)	48	9.87 ^b^

Five RSE concentrations were measured using four rFC and two LAL reagents. The rFC assays are labeled a–d. The results of the actual endotoxin concentrations were transformed into the sample recovery (%) and percentage difference. The average sample recovery (%) and percentage difference were calculated, and the differences between reagents were statistically analyzed using the Kruskal–Wallis H test and Dunn’s test. The significance increased in the following order: a < b < c < d. The same lowercase alphabetical markings indicate that no significant difference occurred between the assays.

**Table 7:** Sample recovery (%) and percentage difference of LAL and rFC assays.

Parameter	Assay	n	Mean
Sample recovery (%)	LAL	146	77.29
	rFC	264	107.91
Percentage difference	LAL	146	24.95
	rFC	264	11.97

Five RSE concentrations were measured using four types of rFC and two types of LAL reagents. All the actual results for each concentration were transformed into the sample recovery (%) and percentage difference. The reagents were divided into two groups: LAL and rFC. The LAL assays included KCA and KTA reagents, whereas rFC assays included PyroGene, Endozyme II, Endozyme II GO, and Endolisa. The average sample recovery (%) and percentage difference between the groups were calculated. Differences between the LAL and rFC assays were statistically analyzed using the Wilcoxon rank-sum test.

**Table 8:** PPC recovery (%) and endotoxin concentrations of recombinant products.

Type			MVD	Dilution Factor		PPC Recovery (%)				Result (EU/mL)			
						rFC		LAL		rFC		LAL	
				rFC	LAL	A	B	KCA	KTA	A	B	KCA	KTA
Antibody therapeutics		Ab1	400	10	10	94.7	113.0	90.0	125.7	<0.001	<0.050	0.001	0.167
		Ab2	400	20	20	111.4	94.7	73.7	67.7	<0.100	<0.100	<0.100	<0.100
		Ab3	4000	50	50	77.6	98.0	75.3	85.0	<0.150	<0.150	<0.150	<0.150
		Ab4	32	10	10	84.8	116.7	85.0	134.7	<0.001	<0.050	0.001	0.001
		Ab5	500	10	10	95.0	115.0	74.0	94.0	<0.050	<0.050	<0.050	<0.072
		Ab6	200	50	50	93.5	102.3	68.0	89.0	<0.050	<0.250	<0.050	<0.075
Recombinant protein	Hormone	P1	3340	10	10	103.5	94.7	60.3	75.3	<0.050	<0.050	<0.0005	<0.050
		P2	16,000	150	150	60.2	55.3	63.3	58.3	<0.750	<0.750	<0.750	<0.750
		P3	16,000	10	10	65.3	101.7	82.7	90.7	<0.001	<0.050	<0.001	0.001
		P4	16,000	100	100	62.7	119.7	97.7	93.7	<0.000	<0.500	<0.000	<0.000
		P5	16,000	10	10	91.9	92.0	75.3	88.0	<0.050	<0.050	<0.0005	<0.050
	Cytokine	P6	50	10	10	95.1	124.7	76.7	128.0	<0.050	<0.050	<0.050	<0.089
		P7	4000	20	20	95.0	105.7	68.0	62.7	<0.100	<0.100	<0.100	<0.100
	Blood coagulation factor	P8	200	10	10	92.8	136.0	80.3	83.7	<1.5	<1.5	0.32	0.36

The recombinant products were diluted with endotoxin-free water to below the maximum effective dilution (MVD). BET was performed on the diluted samples using the LAL and rFC assays, and the PPC recovery (%) was calculated.

**Table 9:** PPC recovery (%) and endotoxin concentrations of toxin products.

Type		MVD	Dilution Factor		PPC Recovery (%)				Result (EU/mL)			
					rFC		LAL		rFC		LAL	
			rFC	LAL	A	B	KCA	KTA	A	B	KCA	KTA
Toxin products	T1	350	10	10	90.0	127.0	84.3	84.3	<0.001	<0.050	<0.050	<0.065
	T2	200	10	10	92.4	82.7	85.3	83.0	<0.001	<0.050	<0.050	<0.050

Toxin products were diluted below the maximum effective dilution (MVD) with endotoxin-free water. BET was performed on the diluted samples using the LAL and rFC assays, and the PPC recovery (%) was calculated.

**Table 10:** PPC recovery (%) and endotoxin concentrations of vaccines.

**Type**		**MVD**	**Dilution Factor**				**PPC Recovery (%)**				**Result (EU/mL)**			
			**rFC**		**LAL**		**rFC**		**LAL**		**rFC**		**LAL**	
			A	B	KCA	KTA	**A**	**B**	**KCA**	**KTA**	**A**	**B**	**KCA**	**KTA**
Bacterial vaccine	BV1	2500	10	10	10	10	59.0	72.3	71.7	119.0	<0.05	<0.05	0.09	0.10
	BV2	5000	10	10	10	10	101.8	122.0	92.3	144.7	<0.05	<0.05	0.12	0.09
	BV3	5000	50	50	50	50	106.9	107.3	89.3	114.0	<0.25	<0.25	0.25	0.37
	BV4	1000	200	300	50	50	56.6	55.7	80.0	143.0	<1.0	<1.5	0.36	5.56
	BV5	800	50	50	50	50	53.3	59.0	65.7	88.7	<0.25	<0.25	0.28	0.46
	BV6	40,000	400	400	200	200	68.3	55.7	90.0	90.7	<2.0	<2.0	1.79	1.20
	BV7	40,000	100	100	100	100	60.1	57.7	88.3	89.7	<0.5	0.75	6.55	11.2
Virusvaccine	VV1	40,000	10	10	10	10	107.6	112.3	77.0	126.7	6.0	1.6	0.19	0.10
	VV2	2000	300	300	100	100	57.2	97.0	55.3	62.0	<1.5	<1.5	<0.5	0.53
	VV3	2000	1000	1000	100	100	68.0	89.0	63.7	54.7	<5.0	<5.0	0.52	0.67
	VV4	400	400	300	100	100	54.3	66.7	54.3	89.0	<2.0	<1.5	0.06	0.18
	VV5	400	400	300	10	10	53.8	58.3	56.3	64.7	<2.0	<1.5	0.05	0.11
	VV6	800	300	300	50	50	82.3	91.3	90.3	105.3	<1.5	<1.5	0.32	0.36
	VV7	2000	40	40	40	40	101.3	104.7	75.7	86.3	<0.2	<0.2	<0.2	0.29

The vaccines were diluted with endotoxin-free water to below the maximum effective dilution (MVD). BET was performed on the diluted samples using the LAL and rFC assays, and the PPC recovery (%) was calculated.

## Data Availability

The data presented in this study are available on request from the corresponding author.

## References

[1] Schneier M., Razdan S., Miller A.M., Briceno M.E., Barua S. (2020). Current technologies to endotoxin detection and removal for biopharmaceutical purification. Biotechnol. Bioeng..

[2] Franco E., Gracia-Recio V. (2018). Endotoxins from a Pharmacopoeial Point of View. Toxins.

[3] Muta T., Oda T., Iwanaga S. (1993). Horseshoe crab coagulation factor B. A unique serine protease zymogen activated by cleavage of an Ile-Ile bond. J. Biol. Chem..

[4] Ding J.L., Ho B. (2010). Endotoxin detection—From *Limulus* amebocyte lysate to recombinant factor C. Endotoxins: Structure, Function and Recognition.

[5] Kawabata S., Koshiba T., Shibata T. (2009). The lipopolysaccharide-activated innate immune response network of the horseshoe crab. Invertebr. Surviv. J..

[6] Perdomo-Morales R., Pardo-Ruiz Z., Spreitzer I., Lagarto A., Montag T. (2011). Monocyte activation test (MAT) reliably detects pyrogens in parenteral formulations of human serum albumin. ALTEX-Altern. Anim. Exp..

[7] Krisfalusi-Gannon J., Ali W., Dellinger K., Robertson L., Brady T.E., Goddard M.K.M., Tinker-Kulberg R., Kepley C.L., Dellinger A.L. (2018). The role of horseshoe crabs in the biomedical industry and recent trends impacting species sustainability. Front. Mar. Sci..

[8] Ding J.L., Chai C., Pui A.W.M., Ho B. (1997). Expression of full length and deletion homologues of *Carcinoscorpius rotundicauda* factor C in *Saccharomyces cerevisiae*: Immunoreactivity and endotoxin binding. J. Endotoxin Res..

[9] Pui A.W.M., Ho B., Ding J.L. (1997). Yeast recombinant factor C from horseshoe crab binds endotoxin and causes bacteriostasis. J. Endotoxin Res..

[10] Roopashree S.D., Ho B., Ding J.L. (1996). Expression of *Carcinoscorpius rotundicauda* factor C in *Pichia pastoris*. Mol. Mar. Biol. Biotechnol..

[11] Maloney T., Phelan R., Simmons N. (2018). Saving the horseshoe crab: A synthetic alternative to horseshoe crab blood for endotoxin detection. PLoS Biol..

[12] Ding J.L., Ho B. (2001). A New era in pyrogen testing. Trends Biotechnol..

[13] Loverock B., Simon B., Burgenson A., Baines A. (2010). A recombinant factor C procedure for the detection of gram-negative bacterial endotoxin. Pharmacopeial Forum.

[14] Abate W., Sattar A.A., Liu J., Conway M.E., Jackson S.K. (2017). Evaluation of recombinant factor C assay for the detection of divergent lipopolysaccharide structural species and comparison with *Limulus* amebocyte lysate-based assays and a human monocyte activity assay. J. Med. Microbiol..

[15] Bolden J., Smith K. (2017). Application of recombinant factor C reagent for the detection of bacterial endotoxins in pharmaceutical products. PDA J. Pharm. Sci. Technol..

[16] Muroi M., Ogura N., Mizumura H., Aketagawa J., Oda T., Tanamoto K.I. (2019). Application of a recombinant three-factor chromogenic reagent, PyroSmart, for bacterial endotoxins test filed in the pharmacopeias. Biol. Pharm. Bull..

[17] Piehler M., Roeder R., Blessing S., Reich J. (2020). Comparison of LAL and rFC assays-Participation in a proficiency test program between 2014 and 2019. Microorganisms.

[18] (2021). European Pharmacopoeia.

[19] Tsuchiya M. (2019). Mechanism of low endotoxin recovery caused by a solution containing a chelating agent and a detergent. Immunome Res..

[20] Schneider C. (2016). Overcoming low endotoxin recovery. Pharm. Technol. Eur..

[21] Schwarz H., Gornicec J., Neuper T., Parigiani M.A., Wallner M., Duschl A., Horejs-Hoeck J. (2017). Biological activity of masked endotoxin. Sci. Rep..

[22] Reich J., Lang P., Grallert H., Motschmann H. (2016). Masking of endotoxin in surfactant samples: Effects on *Limulus*-based detection systems. Biologicals.

[23] Park C.Y., Jung S.H., Bak J.P., Lee S.S., Rhee D.K. (2005). Comparison of the rabbit pyrogen test and *Limulus* amoebocyte lysate (LAL) assay for endotoxin in hepatitis B vaccines and the effect of aluminum hydroxide. Biologicals.

[24] McCullough K.Z., Parenteral Drug Association (2011). The Bacterial Endotoxins Test: A Practical Approach.

[25] Fujita Y., Tokunaga T., Kataoka H. (2011). Saline and buffers minimize the action of interfering factors in the bacterial endotoxins test. Anal. Biochem..

